# Plasma Proteomic Signatures of Glucose Metabolism Disturbances and Early Diabetes

**DOI:** 10.3390/ijms27093844

**Published:** 2026-04-26

**Authors:** Natalia Zieleniewska, Jacek Jamiołkowski, Anders Malarstig, Klev Diamanti, Małgorzata Chlabicz, Marcin Kondraciuk, Kerhan Woo, Irina Kowalska, Karol Kamiński

**Affiliations:** 1 Department of Population Medicine and Lifestyle Diseases Prevention, Medical University of Białystok, Waszyngtona 15B Street, 15-269 Bialystok, Poland; 2 Department of Cardiology and Internal Medicine with Cardiac Intensive Care Unit, Medical University of Białystok, Waszyngtona 15B Street, 15-269 Bialystok, Poland; 3 Department of Medical Epidemiology and Biostatistics, Karolinska Institute, Nobels Road 12A, 171 65 Stockholm, Sweden; 4 Olink, Part of Thermo Fisher Scientific, Salagatan 16A, 753 30 Uppsala, Sweden; 5 Department of Invasive Cardiology and Internal Medicine with Cardiac Intensive Care Unit, Medical University of Białystok, Waszyngtona 15B Street, 15-269 Bialystok, Poland; 6 Department of Internal Medicine and Metabolic Diseases, Medical University of Białystok, Waszyngtona 15B Street, 15-269 Bialystok, Poland

**Keywords:** prediabetes, diabetes type 2, biomarkers, proteomic profiling

## Abstract

Postprandial variability in glucose and protein levels is one of the elements of insulin resistance (IR) and prediabetes, which is an area precursor to type 2 diabetes mellitus (DM). The objective of the study was a comprehensive proteomic analysis according to glucose tolerance in the general population who did not self-report DM or other diseases. We used Olink^®^ Reveal, a novel, high-throughput platform by Olink Proteomics based on their Proximity Extension Assay (PEA), to identify levels of 1034 circulating proteins in small volumes (4 µL) of plasma samples. The study enrolled 508 participants (mean age 52 ± 10.5 years, 47.2% men) from the population-based study, Bialystok PLUS Polish Longitudinal University Study. The study population was categorized according to glucose metabolism in comparison to impaired fasting blood glucose (IFG), impaired glucose tolerance (IGT), and newly diagnosed DM. Analysis of variance (ANOVA) adjusted for age, weight, fat mass, lean mass, and body mass index (BMI), identified 19 proteins significantly associated with categories of glucose tolerance. Of the five markers with the greatest ability to distinguish newly diagnosed diabetes from non-diabetic participants, paralemmin 2 performed best (AUC = 0.81; 77% sensitivity, 75% specificity), whereas furin was the most accurate for detecting any abnormal glucose regulation (AUC = 0.69). A linear regression model adjusted for the same confounding factors showed statistically significant associations between Hb_A1c_ levels and 37 proteins. Our findings highlight multiple proteins with significantly different levels across categories of glucose tolerance, especially between the healthy controls and the group with newly diagnosed DM. The consistent patterns of protein level differences, independent of body composition, suggest potential involvement in the progression of glucose metabolism disturbances and provide unique insights into pathomechanisms. These findings identify PALM2, FURIN, PDZK1, ACAA1, and IL18R1 as potential biomarkers of early dysglycemia.

## 1. Introduction

Omics technologies have revolutionized biomedical research, providing high-throughput, comprehensive methods for studying complex biological systems from small sample volumes (4 µL). These methods allow for a holistic understanding of biological systems and disease mechanisms [1]. Proteomics has emerged as a critical tool for elucidating the molecular mechanisms underpinning various diseases, including diabetes (DM) and atherosclerotic cardiovascular disease (ASCVD) [1]. By enabling the simultaneous quantification and characterization of thousands of proteins, proteomics provides insights into dynamic cellular processes, post-translational modifications, and biomarker discovery [2].

Worldwide, the prevalence of DM is projected to reach 592 million by 2035 [3]. Although there has been increased public awareness, numerous cases remain undiagnosed. Identifying new biological biomarkers for insulin resistance (IR) and DM is essential to improve risk prediction and deepen the understanding of underlying pathways beyond well-known factors. In recent years, advances in omics research have identified potential DM biomarkers, including circulating microRNAs or branched amino acids [4]. Deacylated and acylated ghrelin have emerged as candidate biomarkers for IR [5]. Likewise, serum retinol-binding protein 4 has been shown to contribute to IR in obesity and DM [6]. However, the newly circulating molecules were not included in the scales used for risk stratification. Therefore, a method that quantifies multiple proteins in a single sample may be an alternative for assessing the increase in IR risk.

Importantly, multi-omics analysis may also be vital in identifying proteins involved in the initial complications linked to glucose metabolism disorders. Vascular complications often develop gradually, and early detection could enable more effective preventive measures. Understanding these biomarkers may help develop targeted therapies, ultimately lowering morbidity and mortality related to diabetes-associated cardiovascular diseases (CVDs).

The objective of the study was to conduct a comprehensive proteomic analysis of plasma samples focused on disturbances in glucose metabolism, using Olink^®^ Reveal, a novel high-throughput platform based on Proximity Extension Assay (PEA) technology. Our objective was to identify early biomarkers associated with complications in DM and prediabetes. Specifically, we aimed to develop a panel of proteins that could assist in diagnosing glucose metabolism disorders. By incorporating proteomic profiling into large-scale epidemiological studies, researchers may gain deeper insights into the biological mechanisms linking postprandial glucose fluctuations with long-term health outcomes.

## 2. Results

### 2.1. Clinical Characteristics at Baseline

The study included 508 individuals who did not self-report having DM and other serious diseases, with a mean age of 52 ± 10.5 years, and 47.2% of whom were male. In Table 1, we briefly characterized the study group with respect to glucose metabolism and gender. The average age steadily increased across different groups of glucose metabolism disturbances. Participants with newly diagnosed DM were notably older. BMI and weight showed a similar trend, increasing significantly with impaired glucose metabolism. We noted that fat mass elevates gradually across groups, while lean mass remains relatively stable. Proteomics measurements exhibited very high detectability (mean 79%; median 99%) and precision (mean intra- and interplate coefficients of variation of 7.4% and 7.6%, respectively).

**Figure 1:**
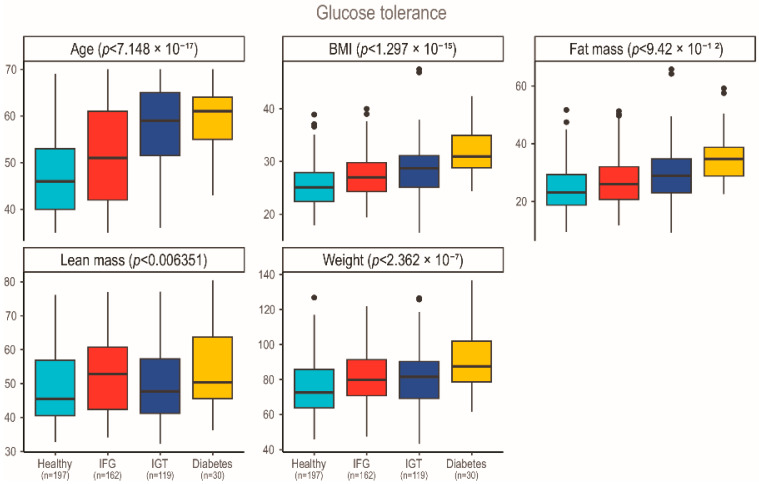
Comparison of glucose tolerance groups across anthropometric and age-related variables. Lean and fat mass are densitometric parameters. *p*-values were extracted from linear regression models that assessed the association between continuous anthropometric or age-related variables (plot facets) and a discrete variable describing glucose tolerance (*x*-axis); one model was fit for each such variable, for a total of 5 models. Titles of each plot indicate the title of the *y*-axis of each facet.

Figure 1 confirms the relationship between glucose metabolism disturbances and continuous clinical variables. There was a strong association between glucose tolerance and age, BMI, weight, and fat mass. Lean mass was also statistically significant, but of a lesser degree than fat mass. These findings underscore the importance of anthropometric and age-related factors in glucose metabolism and the progression of IR and DM and were therefore considered as confounding variables in subsequent models.

**Figure 2:**
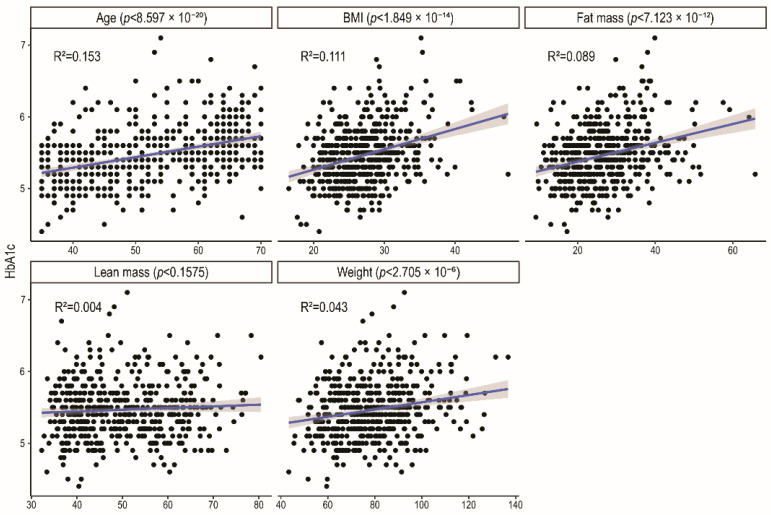
The association between Hb_A1c_ levels and clinical parameters. *p*-values were extracted from linear regression models that assessed the association between continuous anthropometric or age-related variables (plot facets) and HbA1c measurements (*y*-axis); one model was fit for each anthropometric or age-related variable, for a total of 5 models. Titles of each plot indicate the title of the *x*-axis of each facet.

Figure 2 presents the relationships between Hb_A1c_ levels and clinical continuous variables. We observed a strong, significant association between Hb_A1c_ concentration and age, BMI, weight, and fat mass. This analysis shows that adiposity and age are key factors affecting long-term glucose management.

### 2.2. Changes in the Plasma Protein According to Glucose Metabolism

**Figure 3:**
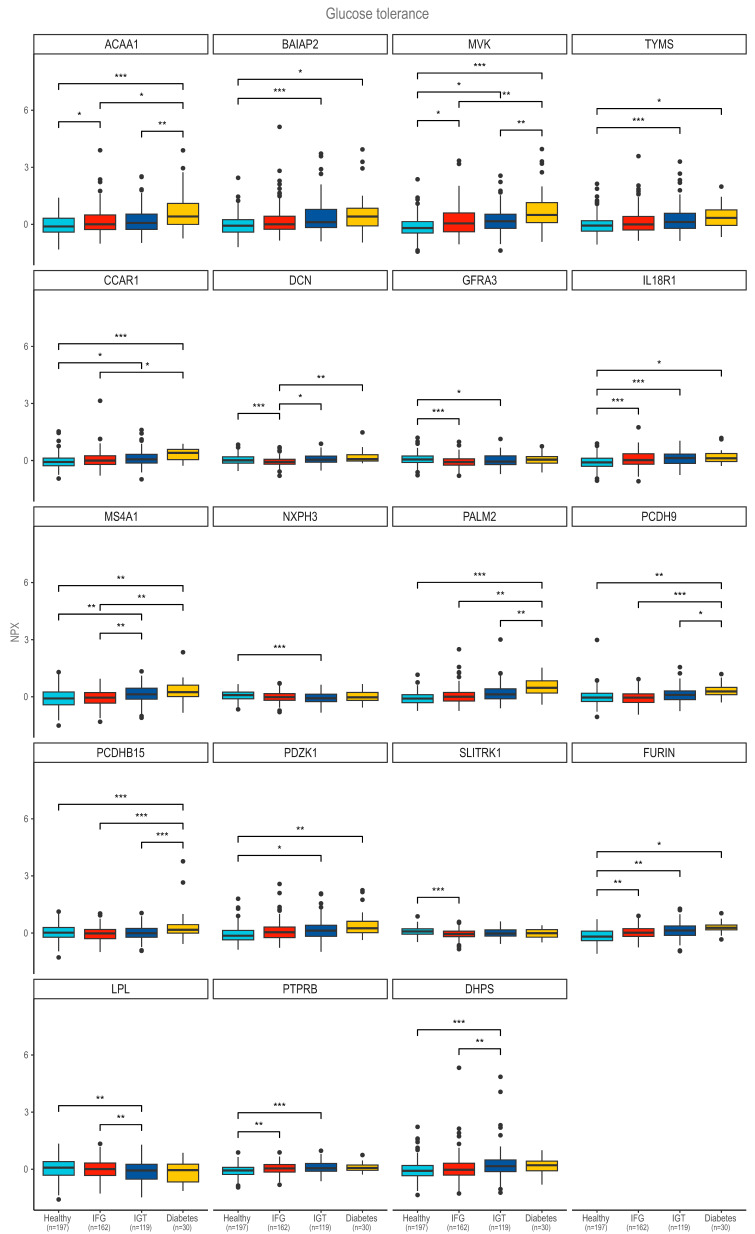
The normalized protein expression across glucose tolerance categories. In the post hoc analysis, statistically significant differences between groups were marked with *, **, and ***. * *p* < 0.05, ** *p* < 0.01, *** *p* < 0.001.

To investigate the associations between glucose metabolism and proteomic profiles, we applied one-way ANOVA models to each of the 1050 quantified proteins. Glucose metabolism status was treated as four groups (normal glucose tolerance, IFG, IGT, and diabetes), and models were adjusted for age, weight, fat mass, lean mass, and BMI. Following multiple-testing correction using the Benjamini–Hochberg method, 19 proteins showed statistically significant associations (adjusted *p* < 0.05) and were retained as potential markers associated with glucose tolerance categories. Subsequently, post hoc analyses were performed to identify specific glucose metabolism disturbances that differed significantly in protein expression. Figure 3 illustrates the NPX levels of the 19 significant proteomic assays across various glucose metabolism groups. A general trend toward higher protein expression in individuals with newly diagnosed DM than in those without impaired glucose metabolism was observed. Many proteins exhibited statistically significant differences in expression across glucose tolerance categories, suggesting their potential relevance in glucose metabolism regulation or as biomarkers of glucose intolerance progression. Specifically, the expression of five proteins: 3-ketoacyl-CoA thiolase peroxisomal (ACAA1), mevalonate kinase (MVK), cell division cycle and apoptosis regulator protein 1 (CCAR1), Protocadherin beta-15 (PCDHB15) and Na(+)/H(+) exchange regulatory cofactor NHE-RF3 (PDZK1) showed strong statistically significant differences between individuals with newly diagnosed DM and population without impaired glucose metabolism. Additionally, decorin (DCN) and GDNF family receptor alpha-3 (GFRA3) expression significantly differed between the healthy population and those with IFG. The expression of SLIT and NTRK-like protein 1 (SLITRK1) varied exclusively between the healthy population and individuals with IFG. Additionally, differences were observed in the expression of lipoprotein lipase (LPL), receptor-type tyrosine-protein phosphatase beta (PTPRB), deoxyhypusine synthase (DHPS), thymidylate synthase (TYMS), and brain-specific angiogenesis inhibitor 1-associated protein 2 (BAIAP2) between the healthy population and those with IGT.

We observed a statistically significant difference in neurexophilin-3 (NXPH3) protein expression only between the group without impaired glucose metabolism and the IGT group. Among the identified proteins, interleukin 18 receptor 1 (IL18R1) was the only one to show significant differences in expression between the healthy population and both the IFG and IGT subpopulations, suggesting its potential as a biomarker of prediabetes. Supplementary Tables S1 and S2 show the results of the ROC analysis. Five proteins achieved an AUC > 0.70 for discriminating newly diagnosed DM from non-diabetic individuals (Supplementary Table S1), with paralemmin 2 (PALM2) showing the strongest performance (AUC = 0.81, 77% sensitivity, 75% specificity). In contrast, discrimination of any abnormal glucose regulation was more modest; the best single marker (FURIN) yielded an AUC of 0.69 (Supplementary Table S2). These findings suggest that individual plasma proteins can separate overt diabetes reasonably well but are less powerful for the earlier dysglycemic states when used in isolation. Combining multiple markers or integrating clinical covariates may therefore be required to improve the detection of abnormal glucose regulation.

### 2.3. The Evaluation of the Association Between Hb_A1c_ and Protein Expression

**Figure 4:**
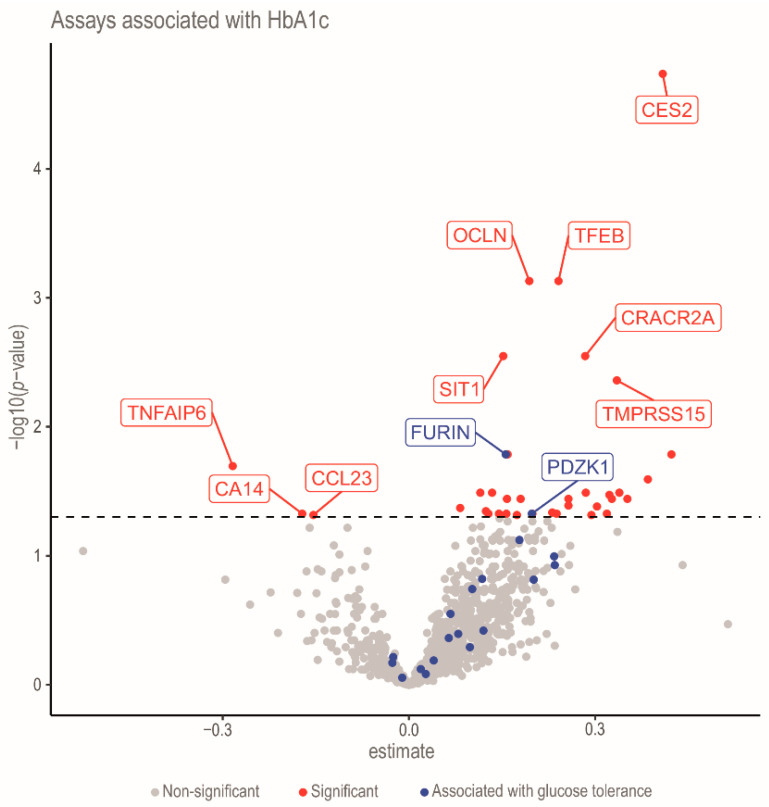
The volcano plot of proteomic associations with Hb_A1c_. Summarizing the results from the linear regression model for Hb_A1c_.

A regression analysis was conducted to evaluate the associations between Hb_A1c_ levels and protein expression (Figure 4). The results highlighted 37 significant associations between Hb_A1c_ and proteins. Among these, notable proteins included cocaine esterase (CES2), transcription factor EB (TFEB), occludin (OCLN), EF-hand calcium-binding domain-containing protein 4B (CRACR2A), signaling threshold-regulating transmembrane adapter 1 (SIT1), enteropeptidase (TMPRSS15), tumor necrosis factor-inducible gene 6 protein (TNFAIP6), carbonic anhydrase 14 (CA14), and C-C motif chemokine 23 (CCL23). Proteins such as CES2, TFEB, OCLN, CRACR2A, SIT1, and TMPRSS15 were associated with increases in Hb_A1c_ levels and impaired glycemic control. Several proteins, including sialic acid-binding Ig-like lectin 7 (SIGLEC7), receptor-interacting serine/threonine-protein kinase 3 (RIPK3), and hematopoietically expressed homeobox protein (HHEX), exhibited strong positive correlations with Hb_A1c_, reinforcing their potential as biomarkers of poor glycemic control (Supplementary Figure S1). Conversely, proteins including TNFAIP6, CA14, CCL23 and arginase-1 (ARG1) demonstrated inverse associations with Hb_A1c_, suggesting a possible regulatory or protective mechanism in glucose metabolism. Moreover, FURIN and PDZK1, the two proteins identified as being associated with glucose tolerance, are also significantly associated with HbA1c concentrations, suggesting their potential involvement in long-term glycemic regulation. These findings highlight proteins significantly associated with Hb_A1c_ levels, underscoring their involvement in glycemic regulation and their potential relevance as biomarkers for DM progression. Notably, proteins like CES2, CRACR2A, and TNFAIP6 emerge as promising candidates for further exploration in the context of diabetes pathophysiology. These results contribute to a deeper understanding of the molecular factors involved in the regulation of or affected by disturbances in glucose metabolism. In addition, we demonstrated notable differences among the proteins in relation to fasting glucose, 2-h glucose, and glycemic excursions. Several proteins remained significantly associated with fasting glucose after adjustment for confounders and correction for multiple testing. The strongest positive associations were observed for MVK (β = 0.015, FDR < 0.001) and PDZK1 (β = 0.011, FDR < 0.001), whereas TNFSF12 showed a negative association (β = −0.006, FDR = 0.008). The gene set enrichment analysis of Hb_A1c_-associated pathways is demonstrated in Supplementary Figure S2. The analysis identifies gene sets that are significantly enriched and functionally categorized based on their normalized enrichment score (NES), as visualized by horizontal bar plots. Notably, strong enrichment was observed in gene sets associated with the immune system, such as cytokine signaling, Toll-like receptor cascades, and interferon signaling, suggesting a potential link between immune pathways and elevated HbA1c levels. Additional enrichment is evident in signal transduction pathways (GPCR and MAPK signaling), cell cycle regulation, and metabolic pathways, particularly those related to RNA and protein metabolism. Furthermore, Supplementary Figure S2 presents a STRING network of the 19 proteins related to glucose tolerance. The set of 19 proteins with an identified relationship with glucose tolerance was queried in STRING v12.0. The network contained all 19 nodes but only 4 unique edges (expected 2), yielding a non-significant PPI enrichment *p*-value of 0.103. No Gene Ontology, KEGG or Reactome terms passed FDR < 0.05. These findings indicate that the proteins do not form a single, tightly interconnected functional module.

## 3. Discussion

Our study identified 19 proteins significantly associated with glucose tolerance independently of body composition, revealing higher protein expression levels in individuals with newly diagnosed DM compared to those without glucose metabolism impairments. Among these, 5 proteins, including ACAA1, exhibited statistically significant differences in expression between individuals with newly diagnosed DM and healthy controls, while IL18R1 emerged as a potential biomarker for prediabetes, showing differential expression in both IFG and IGT groups. ROC analysis showed that several of these markers, most notably PALM2 (AUC = 0.81 for newly-diagnosed DM) and FURIN (AUC = 0.69 for abnormal glucose regulation), possess measurable discriminatory power even when evaluated individually. Further regression analyses identified 37 proteins significantly associated with Hb_A1c_ levels independently of body composition, with notable examples such as CES2 and TNFAIP6 exhibiting positive and inverse correlations, respectively, suggesting distinct roles in glycemic regulation. Proteins such as SIGLEC7 and RIPK3 showed strong positive associations with Hb_A1c_, reinforcing their potential utility as biomarkers of poor glycemic control, whereas ARG1 demonstrated a negative correlation, indicating a possible protective mechanism. The proteins that significantly differed in postprandial expression were involved in numerous pathways, particularly in innate and adaptive immunity, cell cycle regulation, and metabolism. These findings provide valuable insights into the molecular determinants of glucose metabolism and Hb_A1c_ variability, highlighting key proteins with potential implications for understanding and managing DM progression. The comprehensive enrichment profile highlights the multifaceted biological processes potentially modulated in individuals with elevated HbA1c, suggesting that dysregulation extends beyond glucose metabolism to include immune activation, signal transduction, and transcriptional control.

According to our findings, five proteins, FURIN, PALM2, PDZK1, ACAA1, and IL18R1, demonstrated statistically significant associations and emerged as sensitive biomarkers for newly diagnosed DM and abnormal glucose regulation. Notably, the FURIN and PDZK1 levels correlated with glucose tolerance metrics and were significantly associated with HbA1c concentrations, suggesting involvement in longer-term glycemic regulation and underscoring their clinical relevance as early-stage dysglycemia biomarkers. Beyond glucose metabolism, both proteins have been linked to broader cardiometabolic risk. FURIN has been associated with hypertension, atherosclerosis, and increased cardiovascular risk, likely via the activation of substrates involved in vascular remodeling and inflammation [7,8]. PDZK1, through the regulation of the HDL receptor SR-BI, is essential for cholesterol homeostasis and has been implicated in atherosclerosis and coronary artery disease [9]. IL18R1, via pro-inflammatory signaling, has been studied in obesity-related inflammation and IR, further supporting its relevance to chronic metabolic disorders [10,11]. Although PALM2 remains less well-characterized, its role in cell signaling suggests potential connections to lifestyle-related diseases and warrants investigation in broader pathophysiological contexts [12]. Together, these proteins constitute promising biomarkers at the interface of glucose metabolism and cardiometabolic disease.

Several proteomic studies have proposed potential biomarkers for DM [13,14,15,16,17,18,19], but only a few have examined the associations between proteins and diabetes after rigorously accounting for clinical and anthropometric variables. The main advantage of our study is the use of high-throughput proteomic profiling with the assessment of changes after glucose loading independently of body composition in individuals without diagnosed diabetes or serious comorbidities, which limits the influence of confounding factors related to treatment or advanced disease. Most of the associations remained after multivariate adjustment, supporting the view that changes in circulating protein levels may precede clinical diabetes as defined by standard laboratory criteria and may have prognostic value for diabetes and early vascular complications.

Recent affinity-based and quantitative proteomics studies have shown that selected proteins and multi-marker panels can predict DM, respond to glucose loading, and diagnose prediabetes [20]. However, most prior studies have focused on DM onset rather than the earliest health consequences of dysglycemia [17,18]. In line with evidence that early hyperglycemia-related complications are driven by glycation, inflammation, oxidative stress, and transcriptomic perturbations [21,22,23], our study was designed to relate glucose-metabolism phenotypes to protein-expression changes after glucose loading, thereby providing insight into mechanisms underlying early complications of IR.

Within this framework, PALM2 emerged as a candidate biomarker for newly diagnosed type 2 DM. Although PALM2 has mainly been linked to adverse outcomes in malignancy [24,25,26], its robust association with dysglycemia in our cohort suggests a previously unrecognized role in early DM, warranting mechanistic investigation. FURIN, a pleiotropic proprotein convertase, was the most prominent candidate marker of abnormal glucose regulation and is supported by converging genetic, epigenetic, and proteomic data, implicating it in cardiometabolic risk and DM pathogenesis [26,27].

PDZK1 showed consistent associations with both glucose tolerance and HbA1c. Given its role as a scaffolding protein regulating NHE3 and other transporters in the intestine, kidney, and liver [28,29], and emerging links between NHE3 dysfunction, PDZK1 deficiency, and dysglycemia [9,30], our findings highlight PDZK1 as a potential molecular bridge between epithelial ion transport and glucose homeostasis. We also observed higher ACAA1 expression in newly diagnosed DM, consistent with its central role in peroxisomal β-oxidation and prior evidence connecting ACAA1 to metabolic remodeling and therapeutic modulation of IR and DM [28]. Elevated ACAA1 may reflect early atherosclerotic changes in prediabetes and early DM.

Finally, IL18R1 was selectively increased in individuals with combined IFG and IGT, aligning with the established links between IL-18 signaling, obesity, IR, and dysmetabolic inflammation [31]. Taken together, our findings indicate that PALM2, FURIN, PDZK1, ACAA1, and IL18R1 capture complementary aspects of early dysglycemia, and they support further prospective studies to clarify their utility as biomarkers.

The present study has several limitations that should be considered when interpreting the findings. First, its cross-sectional design captures only a single time point, thereby precluding causal inference, limiting the assessment of temporal relationships, and precluding the evaluation of the predictive performance of the identified proteins. Both glucose metabolism status and proteomic profiles were assessed at a single time point, which may not reflect intra-individual variability or dynamic biological changes over time. Our study includes a single fasting glucose measurement, which could affect glucose-tolerance group classifications and underestimate newly diagnosed DM. The proteomic dataset was generated for hypothesis-generating purposes and provides the relative protein abundance expressed as normalized log2-scaled NPX values rather than absolute concentrations. Moreover, no orthogonal validation was performed. Despite the application of false discovery rate correction, the exploratory character of the analysis and the large number of proteins examined increase the risk of false-positive findings. Finally, although the statistical models were adjusted for key anthropometric variables, residual confounding by unmeasured factors, such as diet and physical activity, cannot be excluded. The exclusion of individuals with previously diagnosed diseases may have introduced selection bias and may limit the generalizability of the results to broader or clinical populations. The strengths of the study include the size of the overall study population and the simultaneous, multidimensional evaluation of proteins across different families and pathways. The value of the work was enhanced by conducting a modern statistical analysis that accounted for protein expression adjusted for clinical parameters. In summary, our data nominate PALM2, FURIN, ACAA1, IL18R, PDZK1, and a subset of additional proteins as plausible biomarkers of early dysglycemia. Prospective validation will be essential to define their predictive value for incident DM and its early vascular complications.

## 4. Materials and Methods

### 4.1. Study Group Characteristics

The cross-sectional, population-based study was conducted from 2018 among participants in the Białystok PLUS study (Poland), which has previously been described in more detail [31]. Briefly, we invited residents of the medium-sized city of Bialystok, aged 20–79 years, who were randomly selected by age and sex to reflect the demographic structure of the general population. Of these, participants aged 35–70 were selected, yielding, after applying exclusion criteria, a group of 508 subjects. Participants with incomplete clinical data were excluded a priori.

The following exclusion criteria were applied: individuals with a history of type 1 and type 2 diabetes, individuals with a history of inflammatory diseases (psoriasis, rheumatoid arthritis, lupus, inflammatory bowel diseases), individuals with a history of cancer, individuals with a history of serious cardiovascular diseases (after myocardial infarction, coronary artery bypass surgery, coronary artery angioplasty, stroke), individuals with a history of Parkinson’s and Alzheimer’s disease, and individuals taking steroidal or immunosuppressive medication.

### 4.2. Data Collection

Information about participant health status and demographic data was collected using standardized questionnaires. Anthropometric measurements were performed by qualified medical personnel. The subjects’ measurements were taken with a SECA 201 tape (SECA, Hamburg, Germany), which enabled measurements of height, waist, and hips. Waist-to-hip ratio (WHR) was declared as the relation between hip and waist circumference. Body mass index (BMI) was calculated individually by dividing a subject’s weight in kilograms by their height in meters squared and is expressed in kg/m^2^. Parameters such as weight, fat, and lean mass were obtained using the InBody 770 (InBody, Eschborn, Germany).

Body composition measurements were performed using dual-energy X-ray absorptiometry (DEXA; Lunar iDXA, GE Healthcare, Madison, WI, USA). Total body mass was divided into three compartments: fat mass, lean mass, and bone mass, which were measured automatically.

The study group was categorized by glucose tolerance. Participants had been fasting for 8 to 12 h before the Oral Glucose Tolerance Test (OGTT) and reported sleeping through the night. Then, 75 g of glucose dissolved in water was administered orally. The subsequent blood sample was collected two hours later. During this period, the participants refrained from any physical activity. The diagnosis of DM or prediabetes was made in accordance with the World Health Organization (WHO) criteria [32]. Newly diagnosed DM was identified based on a 120-min post-glucose load glucose ≥ 200 mg/dL and/or glycated hemoglobin A1c (Hb_A1c_) ≥ 6.5%. The group without impaired glucose metabolism consisted of individuals with fasting glucose < 100 mg/dL and 2-h glucose < 140 mg/dL. We classified individuals in the impaired fasting blood glucose (IFG) group as having fasting glucose between 100–125 mg/dL and post-120 min OGTT glucose < 140 mg/dL. The impaired glucose tolerance (IGT) group consisted of individuals with glycemia ≥ 140 mg/dL at 120 min but <200 mg/dL, regardless of fasting glycemia. We classified IFG and IGT as prediabetes. Due to a single fasting glucose measurement, the patients who did not report a history of DM and had a fasting glucose level above 126 mg/dL and a blood glucose level in the range of 140 to 200 mg/dL after 2 h of oral glucose loading were included in the IGT group, unless the Hb_A1c_ ≥ 6.5%, when the participant was considered diabetic.

The blood samples were then centrifuged and stored at −70 °C until further analysis. Fasting glucose levels and the 120-min glucose levels in OGTT were measured using the enzymatic reference method with hexokinase on the Cobas c111 device (Roche Diagnostics, Meylan, France). The level of Hb_A1c_ was determined using ion-exchange high-performance liquid chromatography on a D 10 device (Bio-Rad, Hercules, CA, USA).

### 4.3. Proteomic Profiling

EDTA plasma samples were stored in a biobank at −70 °C, thawed at 4 °C, prepared, and sent to Olink Proteomics (Uppsala, Sweden) for proteomic analysis. Olink^®^ Reveal offers deep profiling of 1034 proteins, along with three Olink internal control assays (extension, incubation, and amplification control) [33]. This panel covers a broad range of the proteome, including biomarkers involved in inflammation, cellular metabolic processes, cell adhesion, immune response, and complement activation. Protein abundances were measured using the Olink Multiplex Proximity Extension Assay (PEA) with Next Generation Sequencing readout. The platform produces NPX (Normalized Protein eXpression) values—unitless, log2-scaled measures of relative abundance. NPX is obtained by normalizing raw counts to internal extension/amplification controls, aligning plates using inter-plate controls, and centering to the panel reference (overall median). Consequently, values reflect relative (fold) differences between samples (2-fold per 1 NPX) and are not absolute concentrations. The limit of detection (LOD) was estimated as the concentration of the negative controls across all plates plus three standard deviations [34].

### 4.4. Statistical Analysis

External control samples and internal control assays were removed from the Olink data. Four proteins were flagged with warnings by Olink’s run quality control (QC) and were excluded from the downstream analysis, while all 508 processed samples passed. Outlier samples were determined based on large deviations (±3 standard deviations) from the mean of the first two principal components of a principal components analysis (PCA) and similar large deviations from plotting the interquartile range (IQR) versus the sample median NPX across all assays for each sample. Five samples were identified as outliers in both the PCA and the median vs. IQR plots and were removed from downstream analysis.

Covariates for all statistical models were selected based on canonical correlation and regression analyses of clinical/anthropometric variables with the outcome variables (glucose tolerance status and HbA1c).

To identify proteins linked to glucose metabolism categories, we performed a series of one-way ANOVA models, each applied to one of the 1050 quantified proteins, using glucose tolerance status as a four-level categorical independent variable. Each model was adjusted for age, weight, fat mass, lean mass, and BMI. The ANOVA analyses were followed by post hoc pairwise comparisons where relevant. To account for multiple testing, Benjamini–Hochberg false discovery rate correction was applied across all protein-wise ANOVA *p*-values. Proteins with FDR-adjusted *p*-values < 0.05 were considered statistically significant. A separate linear regression model was used to assess the associations between HbA1c and protein levels (NPX values), with covariates including age, weight, fat mass, and BMI. Most analyses were conducted using the R (ver. 4.4.3) package OlinkAnalyze.

Nineteen proteins that differed significantly between glucose-metabolism groups in the one-way ANOVA (FDR < 0.05) were subjected to ROC analysis. NPX values (log2-transformed relative concentrations) served as continuous predictors in two binary classification tasks: (1) DM detection—newly diagnosed type 2 DM vs. all non-diabetic participants (healthy + IFG + IGT), (2) abnormal glucose regulation (AGR) detection—IFG + IGT + DM vs. normoglycemic controls. Receiver operating characteristic (ROC) curves and areas under the curve (AUCs) with 95% confidence intervals were computed with the pROC package (v.1.18.0) in R (ver. 4.4.3). The *p*-values for tests of the AUC against 0.5 were also provided. Optimal cut-off values were defined as the points that minimized the Euclidean distance to the perfect classifier coordinates (0, 1) [35]. Sensitivity and specificity reported in Supplementary Tables S1 and S2 refer to these cut-offs. Moreover, protein–protein associations were evaluated using STRING v12.0 (accessed 15 January 2025) with the 19 human protein symbols, medium confidence (score ≥ 0.40), default evidence channels, and FDR correction for enrichment analyses [36]. No sensitivity analyses were performed because the study was exploratory and used a complete dataset with no missing values. The STROBE guidelines for observational studies were applied in this study.

## 5. Conclusions

Our findings highlight multiple proteins with significantly different levels across glucose tolerance categories, particularly between healthy controls and the group with newly diagnosed DM. The consistent patterns of protein-level differences, independent of body composition, suggest a potential role in the progression of glucose metabolism disturbances and provide insights into pathomechanisms. This population-based proteomic screen identifies a compact panel of plasma proteins that track the progressive worsening of glucose tolerance independently of adiposity.

## Figures and Tables

**Table 1:** Baseline characteristics of the study population with respect to glucose metabolism and gender.

	Population Without Glucose Metabolism Disturbances n = 197	Population with IFG n = 162	Population with IGT n = 119	Population with Newly Diagnosed DM n = 30
Age, years	47.9 ± 9.8	51.7 ± 10.3	57.3 ± 9.4	59.2 ± 7.2
BMI, kg/m^2^	25.5 ± 4.0	27.2 ± 4.1	28.8 ± 4.8	32.0 ± 4.9
Weight, kg	75.2 ± 15.0	80.8 ± 15.5	81.4 ± 16.2	91.4 ± 18.4
Fat mass, kg	23.7 ± 7.4	26.9 ± 8.6	30.1 ± 9.4	35.4 ± 9.3
Lean mass, kg	48.4 ± 9.6	51.3 ± 10.6	49.8 ± 10.5	54.5 ± 13
Hb_A1c_, %	5.3 ± 0.3	5.5 ± 0.3	5.6 ± 0.4	6.1 ± 0.5
Fasting glucose, mg/dL	92.2 ± 5.3	106.5 ± 5.8	106.9 ± 10.9	122.3 ± 19.6
Glucose after 2 h in OGTT, mg/dL	105.9 ± 18.8	113.4 ± 16.7	160.0 ± 15.9	226.4 ± 38.2

IFG—impaired fasting glucose, IGT—impaired glucose tolerance, DM—diabetes mellitus type 2, BMI—body mass index, Hb_A1c_—glycated hemoglobin A1c, OGTT—oral glucose tolerance test. Fat and lean mass parameters based on densitometry measurements. All variables included in the analysis were complete, no missing data.

## Data Availability

The data presented in this study are available on request from the corresponding author.

## Supplementary Materials

The following supporting information can be downloaded at: https://www.mdpi.com/article/10.3390/ijms27093844/s1.
