# Supplementary material for: Plasma Proteomic Signatures of Glucose Metabolism Disturbances and Early Diabetes
Source: Int J Mol Sci. 2026 Apr 26;27(9):3844. doi: 10.3390/ijms27093844 (PMC13163269; doi:10.3390/ijms27093844)
Supplement: Supplementary file 1 [file ijms-27-03844-s001.zip › ijms-4196175-supplementary.pdf]

Supplementary Table S1 Diagnostic performance of the top-five proteins for detecting newly diagnosed diabetes.

Protein	AUC (95% C.I.)	Sensitivity*	Specificity*
PALM2	0.8098 (0.734-0.886)	76.7%	75.3%
CCAR1	0.7580 (0.676-0.840)	70.0%	74.7%
PCDH9	0.7573 (0.678-0.837)	73.3%	69.7%
Furin	0.7393 (0.664-0.814)	80.0%	64.0%
PDZK1	0.7203 (0.633-0.807)	63.3%	71.3%

CCAR1 - cell division cycle and apoptosis regulator protein 1; PALM2 – paralemmin 2 ; PCDH9 – protocatherin 9; PDZK1 – PDZ containing 1.* Sensitivity and specificity were calculated for the threshold that minimised the Euclidean distance to the perfect classifier point (100% sensitivity, 100% specificity) on the ROC curve, as recommended by Song et al., 2014 [13].

Supplementary Table S2 Diagnostic performance of the top-five proteins for detecting abnormal glucose regulation.

Protein	AUC (95% C.I.)	Sensitivity*	Specificity*
Furin	0.6890 (0.641-0.737)	65.9%	65.5%
PALM2	0.6531 (0.605-0.701)	60.8%	63.5%
IL18R1	0.6514 (0.604-0.699)	51.4%	72.6%
PDZK1	0.6505 (0.602-0.699)	60.5%	62.9%

PTPRB	0.6456 (0.597-0.694)	65.6%	55.3%

IL18R1 – interleukin 19 receptor 1; PALM2 – paralemmin 2; PDZK1 – PDZ containing 1; PTPRB - receptor-type tyrosine-protein phosphatase beta. * Sensitivity and specificity were calculated for the threshold that minimised the Euclidean distance to the perfect classifier point (100% sensitivity, 100% specificity) on the ROC curve, as recommended by Song et al., 2014 [13].

Supplementary Figure S1 – Identification of 37 assays significantly associated with Hb_{A1c}.

Visualization of NPX level associations using scatterplots and linear regression lines.

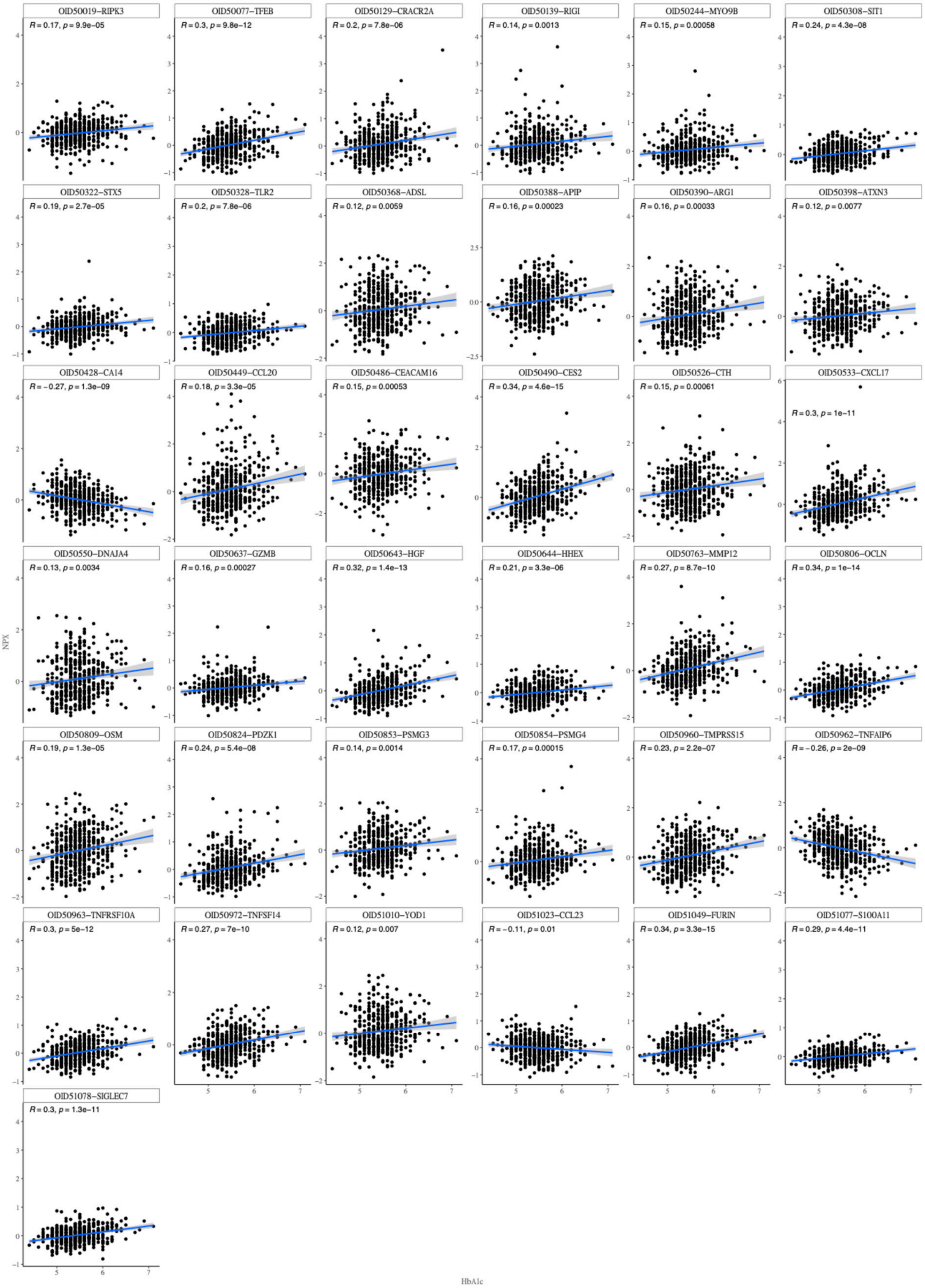

OID50019–RIPK3
R = 0.17, p = 9.9e–05
OID50077–TFEB
R = 0.3, p = 9.8e–12
OID50129–CRACR2A
R = 0.2, p = 7.8e–06
OID50139–RIGI
R = 0.14, p = 0.0013
OID50244–MYO9B
R = 0.15, p = 0.00058
OID50308–SIT1
R = 0.24, p = 4.3e–08
OID50322–STX5
R = 0.19, p = 2.7e–05
OID50328–TLR2
R = 0.2, p = 7.8e–06
OID50368–ADSL
R = 0.12, p = 0.0059
OID50388–APIP
R = 0.16, p = 0.00023
OID50390–ARG1
R = 0.16, p = 0.00033
OID50398–ATXN3
R = 0.12, p = 0.0077
OID50428–CA14
R = –0.27, p = 1.3e–09
OID50449–CCL20
R = 0.18, p = 3.3e–05
OID50486–CEACAM16
R = 0.15, p = 0.00053
OID50490–CES2
R = 0.34, p = 4.6e–15
OID50526–CTH
R = 0.15, p = 0.00061
OID50533–CXCL17
R = 0.3, p = 1e–11
OID50550–DNAJA4
R = 0.13, p = 0.0034
OID50637–GZMB
R = 0.16, p = 0.00027
OID50643–HGF
R = 0.32, p = 1.4e–13
OID50644–HHEX
R = 0.21, p = 3.3e–06
OID50763–MMP12
R = 0.27, p = 8.7e–10
OID50806–OCLN
R = 0.34, p = 1e–14
OID50809–OSM
R = 0.19, p = 1.3e–05
OID50824–PDZK1
R = 0.24, p = 5.4e–08
OID50853–PSMG3
R = 0.14, p = 0.0014
OID50854–PSMG4
R = 0.17, p = 0.00015
OID50960–TMPRSS15
R = 0.23, p = 2.2e–07
OID50962–TNFAIP6
R = –0.26, p = 2e–09
OID50963–TNFRSF10A
R = 0.3, p = 5e–12
OID50972–TNFSF14
R = 0.27, p = 7e–10
OID51010–YOD1
R = 0.12, p = 0.007
OID51023–CCL23
R = –0.11, p = 0.01
OID51049–FURIN
R = 0.34, p = 3.3e–15
OID51077–S100A11
R = 0.29, p = 4.4e–11
OID51078–SIGLEC7
R = 0.3, p = 1.3e–11
NPX
HbA1c

Supplementary Figure S2 – The Gene Set Enrichment Analysis of Hb_{A1c} -associated pathways. Tiles of the heatmap visualize Reactome pathways (left hand side y-axis) enriched based on the effect sizes of proteomics measurements associated with Hb_{A1c}.

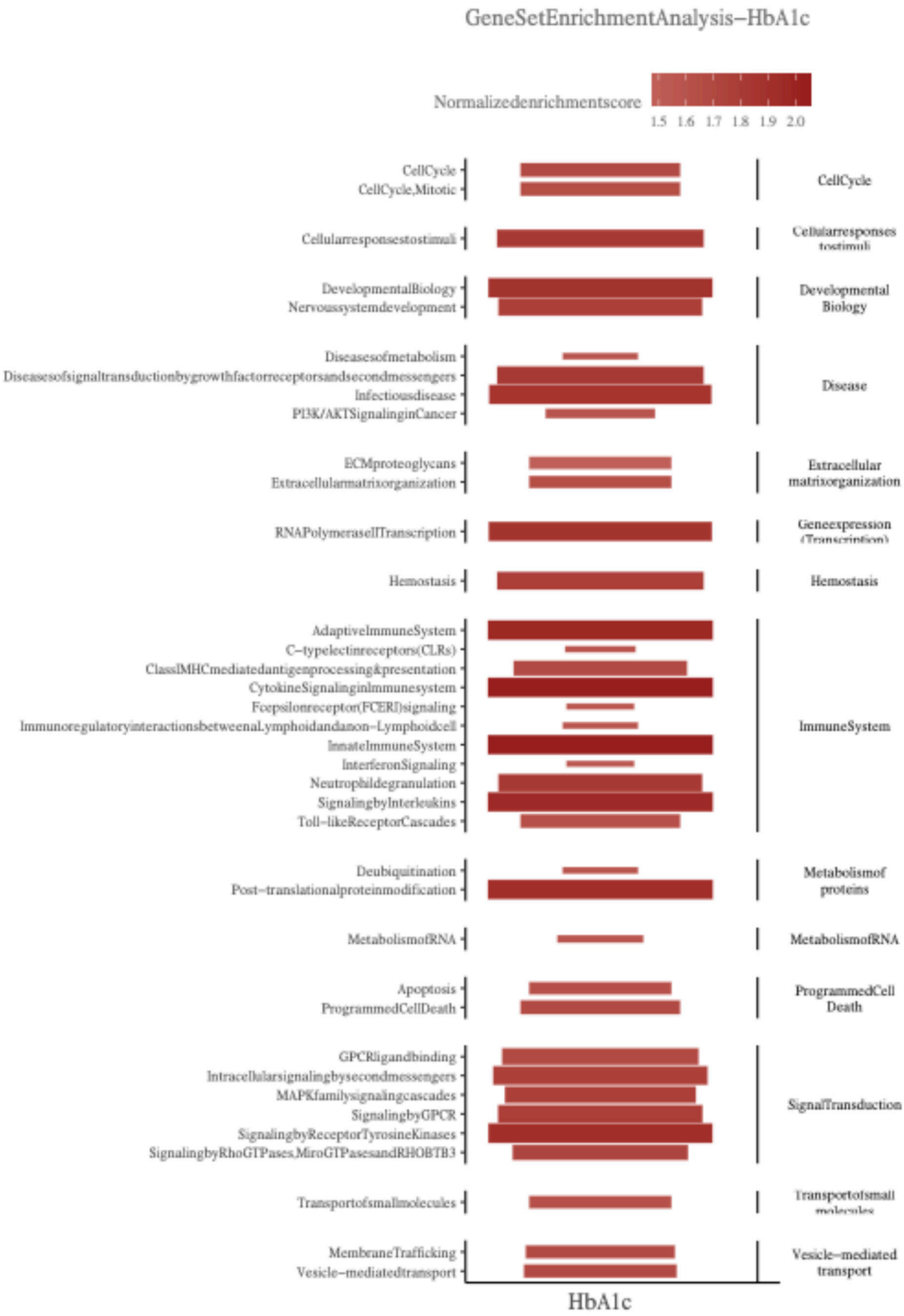

GeneSetEnrichmentAnalysis–HbA1c
Normalizedenrichmentscore
1.5 1.6 1.7 1.8 1.9 2.0
CellCycle
CellCycle,Mitotic
Cellularresponsestostimuli
DevelopmentalBiology
Nervoussystemdevelopment
Diseasesofmetabolism
Diseasesofsignaltransductionbygrowthfactorreceptorsandsecondmessengers
Infectiousdisease
PI3K/AKTSignalinginCancer
ECMproteoglycans
Extracellularmatrixorganization
RNAPolymeraseIITranscription
Hemostasis
AdaptiveImmuneSystem
C–typelectinreceptors(CLRs)
ClassIMHCmediatedantigenprocessing&presentation
CytokineSignalinginImmunesystem
Fcepsilonreceptor(FCERI)signaling
ImmunoregulatoryinteractionsbetweenaLymphoidandanon–Lymphoidcell
InnateImmuneSystem
InterferonSignaling
Neutrophildegranulation
SignalingbyInterleukins
Toll–likeReceptorCascades
Deubiquitination
Post–translationalproteinmodification
MetabolismofRNA
Apoptosis
ProgrammedCellDeath
GPCRligandbinding
Intracellularsignalingbysecondmessengers
MAPKfamilysignalingcascades
SignalingbyGPCR
SignalingbyReceptorTyrosineKinases
SignalingbyRhoGTPases,MiroGTPasesandRHOBTB3
Transportofsmallmolecules
MembraneTrafficking
Vesicle–mediatedtransport
CellCycle
Developmental Biology
Disease
Extracellular matrixorganization
Hemostasis
ImmuneSystem
Metabolismof proteins
MetabolismofRNA
ProgrammedCell Death
SignalTransduction
Vesicle–mediated transport
HbA1c

The size of each tile indicates the -log10(p-value) of each enriched pathway, and intensity of the color of each tile indicates the corresponding normalized enrichment score (NES). The right hand side of the y-axis summarizes the biological functions of the enriched pathways using level 1 pathway from Reactome.

Supplementary Figure S3 - STRING medium-confidence interaction network of 19 proteins related to glucose tolerance (score ≥ 0.40).

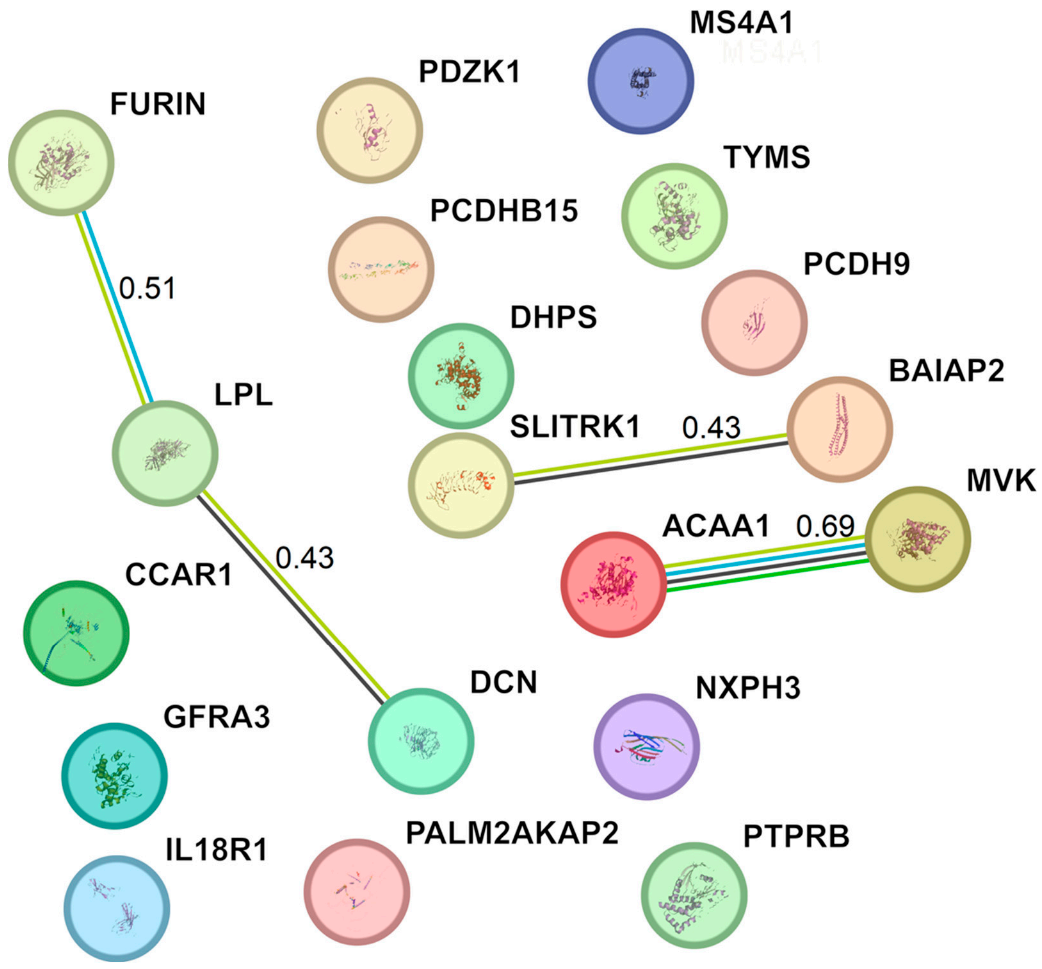

Nodes represent the 19 proteins; edges indicate STRING combined scores ≥ 0.40 (“medium” confidence; STRING v12.0, accessed 19 Jul 2025). Labels on edges give the combined score (0 – 1; 1 = strongest evidence).
